# Improvement of Hemodynamics and Quality of Life Before and After Interatrial Shunt Devices Implantation for Chronic Heart Failure: A Systematic Review and Meta-analysis

**DOI:** 10.2174/011573403X376422250522094942

**Published:** 2025-06-10

**Authors:** Yugen Guan, Lei Yang, Yuwen Lu, Xiaogan Liang, Ruiqi Wang, Rongrong Shen, Liang Yang, Jingwen Song, Shaofei Liu, Yuan Bai, Zhifu Guo, Ni Zhu

**Affiliations:** 1 Department of Cardiology, Changhai Hospital Affiliated to Naval Medical University, Shanghai 200433, China

**Keywords:** Heart failure, interatrial shunt devices, hemodynamics, pulmonary capillary wedge pressure, cardiac devices, dyspnea

## Abstract

**Introduction:**

The objective of this study was to compare the quality of life and hemodynamic changes before and after transcatheter atrial septal shunt implantation.

**Methods:**

A systematic search was conducted in the Cochrane Library, PubMed, and Embase from inception to September 2023 for studies reporting on hemodynamics or quality of life in patients with chronic heart failure after atrial septal shunt implantation. A meta-analysis was performed, in which a total of 1026 participants from 13 articles were included.

**Results and Discussion:**

Following the implantation, pulmonary capillary wedge pressure (PCWP) decreased by 2.60 mmHg. Right atrial pressure (RAP) increased by 1.30 mmHg and left ventricular ejection fraction (LVEF) increased by 2.13%. However, there were no significant differences in cardiac output and mean pulmonary artery pressure (mPAP) after operation. Minnesota Living with Heart Failure (MLWHF) Score decreased by -19.28, while the Kansas City Cardiomyopathy Questionnaire (KCCQ) score increased by 25.41. Moreover, 6-minute walking distance (6MWD) increased by 32.22 m. The results of subgroup analysis showed that for patients with heart failure with preserved ejection fraction (HFpEF) and heart failure with mildly reduced ejection fraction (HFmrEF), LVEF increased by 3.09% while CO increased by 1.01 L/min after operation. Meanwhile, PCWP significantly decreased by 2.67 mmHg and MLWHF scores decreased by 19.28. Additionally, 6MWD significantly increased by 27.5 m. However, there were no significant changes in RAP and mPAP after operation. For patients with heart failure with reduced ejection fraction (HFrEF), interatrial shunt device implantation did not achieve a significant increase in LVEF.

**Conclusion:**

These findings suggest that while atrial septal shunt implantation might not yield LVEF elevation among patients with HFrEF, it improves hemodynamic parameters, exercise endurance, and QoL among individuals with HFpEF/HFmrEF.

## 1 INTRODUCTION

Heart failure (HF) remains a significant global public health issue, which is impacting approximately 1-2% of the global population. It is estimated that over 64 million individuals worldwide are affected by HF [1-5]. This condition is mainly caused by insufficiency and/or abnormal distribution of cardiac output, which leads to a subsequent rise in pulmonary capillary wedge pressure (PCWP) and presents with symptoms of dyspnea on exertion [6-9].

Guideline-directed medical therapy (GDMT) and innovative cardiac devices have significantly enhanced the management of patients diagnosed with heart failure with reduced ejection fraction (HFrEF) [10-14]. However, it is important to note that over 50% of heart failure (HF) patients exhibit left ventricular ejection fraction (LVEF) exceeding 40%, in which HF with LVEF of 40% to 50% has been termed HF with midrange EF (HFmrEF) while LVEF >50% is defined as HF with preserved EF (HFpEF) [15, 16]. Although sodium-glucose cotransporter type 2 inhibitors (SGLT2i) have been proven to reduce HFpEF hospitalization or cardiovascular mortality, the findings from the EMPEROR-Preserved trial indicate persistently high hospitalization rates [17, 18]. Patients with HFpEF/HFmrEF share common characteristics of elevated left atrial (LA) and LV filling pressures during exercise [19, 20]. It has been revealed that HF may occur after atrial septal defect (ASD) device implantation in elderly patients with an existing ASD and left ventricular diastolic dysfunction. Thus existing ASD may act as a decompression mechanism in defined patients [21-23]. Interatrial shunt devices (IASDs) deliver medical implants designed to redirect blood flow between the atria of the heart. There are a few available on the market. Corvia^®^ Atrial Shunt is a device used to treat heart failure with preserved or mid-range ejection fraction (HFpEF, HFmrEF). It helps reduce elevated left atrial pressure (LAP), which is a major contributor to heart failure symptoms [24]. The shunt is placed *via* a catheter to create a passage between the LA and right atrium (RA), allowing blood to flow from the high-pressure LA to the lower-pressure RA [25]. Another device is V-Wave^®^ Ventura^®^ Interatrial Shunt, which facilitates blood shunting across the interatrial septum. It is designed with specialized features like a Nitinol hourglass frame to anchor the shunt in place and an ePTFE encapsulation to limit tissue growth [26]. To this end, it is presumed that IASDs might reduce left atrium pressures (LAPs) and pulmonary venous pressures at the atrial level and improve the quality of life in patients with HF. Indeed, several clinical studies have demonstrated the potential benefits of IASDs in improving exercise intolerance and hemodynamics for patients with HF [25, 27-30]. However, recently published REDUCE-LAP HF II and RELIEVE-HF results indicated that the efficacy of IASDs still remains controversial [24, 31]. In this systematic review and meta-analysis, we evaluate changes in hemodynamics and quality of life (QoL) in patients with HF before and after IASDs implantation, particularly focusing on those with HFpEF/HFmrEF.

## 2 MATERIALS AND METHODS

### 2.1 Data Sources and Retrieval Process

We conducted a thorough search using PubMed database, Embase database, and the Cochrane Register of Controlled Trials to gather relevant articles on HF and IASD. Our search was limited from the establishment of the databases until September 2023, and we used both keywords and MeSH terms to identify relevant studies. A detailed search strategy is shown in supplementary methods. Additionally, we reviewed previously published systematic reviews to identify potential studies for inclusion in our analysis [10]. Our systematic review was registered on PROSPERO as CRD42022366584 and was conducted according to the Preferred Reporting Items for Systematic Reviews and Meta-Analyses (PRISMA) guidelines [32].

### 2.2 Inclusion Criteria and Exclusion Criteria

The literature included in our analysis met the following four criteria: (1) be a randomized controlled trial or observational study, (2) include patients with symptomatic heart failure and NYHA functional class (II, III, and IV), (3) involve IAS devices implantation, and (4) report baseline and follow-up measures that include hemodynamic or quality-of-life changes.

We excluded literature that did not meet the above criteria, studies that were not randomized controlled trials nor observational studies and those that did not provide complete baseline and follow-up data.

### 2.3 Assessment of Study Quality

We used the ROBINS-I tool to evaluate the quality of non-randomized controlled trials included in our analysis. This tool assessed the literature in seven dimensions and classified it into five grades based on the degree of risk of bias [33]. For the assessment of randomized controlled trials, we used the ROB2 tool to assess the risk of bias in studies based on five aspects according to the criteria of the ROB2 risk of bias assessment tool [34, 35].

### 2.4 Data Extraction and Analysis

In this study, we conducted a comprehensive and independent data extraction process to obtain baseline data, including hemodynamics, exercise tolerance, and quality of life from each study. The data extraction was performed by two independent researchers, and any disagreements were resolved by a third researcher. We used meta-analysis to compare the preoperative and postoperative indicators and data analysis was conducted using Review Manager (Version 5.4) and STATA (version 17). Continuous variables were reported using means and standard deviations, and the heterogeneity among the included studies was assessed using I-square and Chi-square tests. A p-value of less than 0.05 was considered statistically significant. The random effects model was used to pool the data [36]. The publication bias of the article was tested by Egger’s test and Begger’s test. To test the robustness of our findings, we performed a sensitivity analysis by changing the statistical model. Finally, we used the method of using preoperative patients as a control group, which has been used in other studies, to compare the effects of atrial septal shunt implantation on hemodynamic indicators in patients with heart failure [37, 38].

## 3 RESULTS

**Fig (1):**
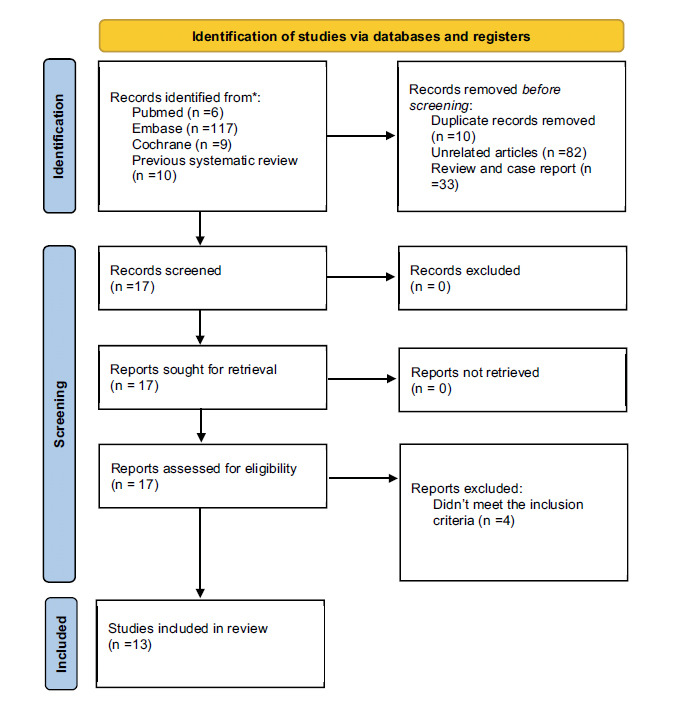
Flow diagram of the study selection process.

After identifying 142 articles relevant to our study topic, we conducted a systematic screening for titles and abstracts and excluded 82 articles from irrelevant papers, as well as 43 duplicates and case reports. From the remaining pool of 17 articles, an additional article was excluded due to the absence of an endpoint index, and three were omitted as they lacked complete data. Finally, 13 articles were included in our study, with a total of 1026 subjects (Fig. **1**) [25, 27-30, 39-45]. Clinical characteristics of selected studies have been provided in Tables **1** and **S1**. There were three randomized controlled trials and ten controlled trials. Upon conducting a rigorous bias assessment, it was observed that none of the chosen articles posed a medium or high risk of bias (Tables **S2a** and **S2b**). Hemodynamic, echocardiographic and QoL outcomes of the included studies are shown in Table **2**.

### 3.1 Hemodynamic and Echocardiographic Changes

**Fig. (2):**
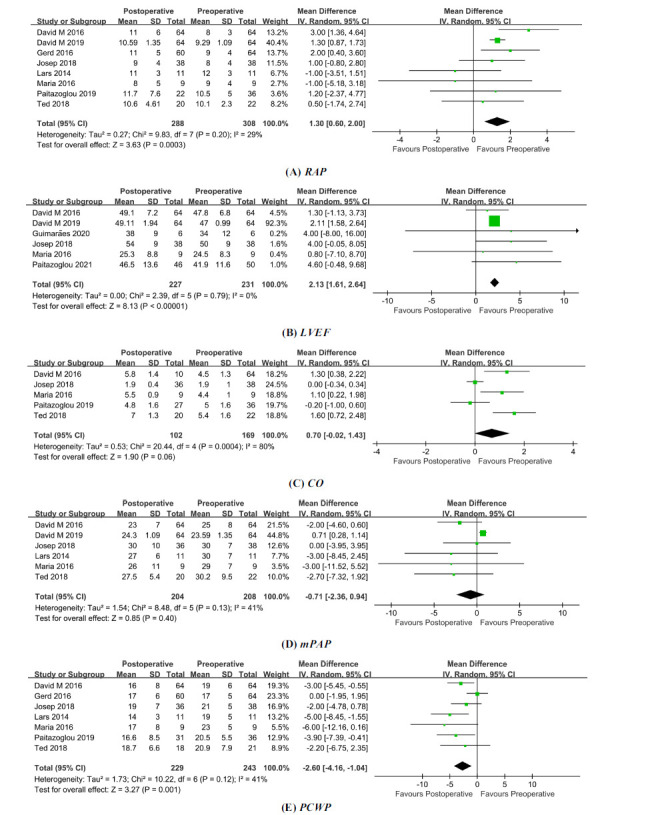
Hemodynamic and echocardiographic changes. Analysis results of (**A**) right atrial pressure (RAP), (**B**) left ventricular ejection fraction (LVEF), (**C**) cardiac output (CO), (**D**) mean pulmonary artery pressure (mPAP), (**E**) pulmonary capillary wedge pressure (PCWP).

Eight articles presented data on right atrial pressure (RAP) both before and after IASD implantation [25, 28, 30, 39, 41, 43-45], while six articles reported on mean pulmonary artery pressure (mPAP) alterations [25, 28, 39, 41, 44, 45]. Furthermore, seven articles examined pulmonary capillary wedge pressure (PCWP) changes [25, 30, 39, 41, 43-45]. Additionally, six articles reported on LVEF changes [27, 28, 39-41, 44], and five articles focused on cardiac output (CO) alterations [25, 39, 41, 43, 44]. The results showed that RAP significantly increased by 1.30 mmHg [95% CI (0.60–2.00); I^2^=29%; *P*<0.0^5^] after IASD implantation (Fig **2A**), while LVEF increased by 2.13% [95% CI (1.61–2.64); I^2^=0%; *P*<0.0^5^] (Fig. **2B**). There were no significant improvements of CO 0.70 L/min [95% CI (-0.02–1.43); I^2^=80%; *P>*0.0^5^] (Fig. **2C**) and mPAP -0.71 mmHg [95% CI (-2.36–0.94)]; I^2^=41%; *P*=0.^40^] (Fig. **2D**), with the reduction of PCWP by 2.60 mmHg [95% CI (-4.16– -1.04); I^2^=41%; *P*<0.0^5^] (Fig. **2E**).

### 3.2 Exercise Endurance and Quality of Life Changes

**Fig. (3):**
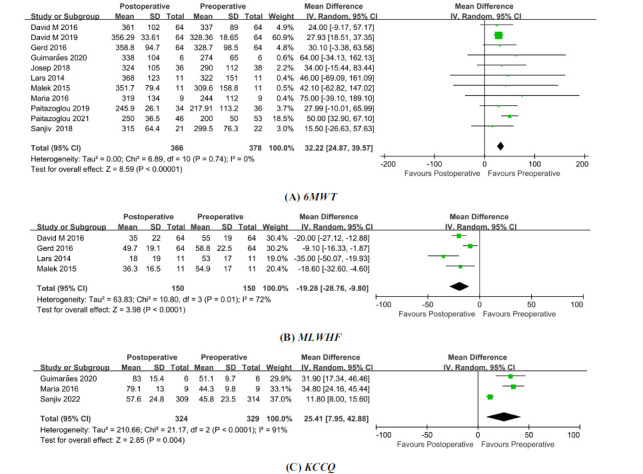
Exercise endurance and quality of life changes. (**A**) Analysis results of 6-minute walk test (6MWT), (**B**) Minnesota Living with Heart Failure (MLWHF) score, (**C**) Kansas City Cardiomyopathy Questionnaire (KCCQ) score.

Data from eleven studies relevant to 6-minute walk test (6MWT) both prior and post to IASD implantation were analyzed [27-30, 39-45]. The analysis revealed a notable improvement of 6MWT -post-procedure, with a significant increase of 32.22 meters [95% CI (24.87–39.57); I^2^=0%; *P*<0.000^1^] (Fig. **3A**). Moreover, four studies presented data on the Minnesota Living with Heart Failure (MLWHF) score before and after IASD implantation [30, 41, 42, 45]. The combined outcomes revealed a significant reduction of MLWHF score by 19.28 [95% CI (-28.76 – -9.80); I^2^=72%; *P*<0.0^5^] (Fig. **3B**). Additionally, three studies presented data on Kansas City Cardiomyopathy Questionnaire (KCCQ) score [24, 39, 40]. The outcomes revealed a significant increase in KCCQ score by 25.41 [95%CI (7.95, 42.88); I^2^=91%; *P*<0.0^5^] (Fig. **3C**).

### 3.3 Results of Subgroup Analysis

**Fig. (4):**
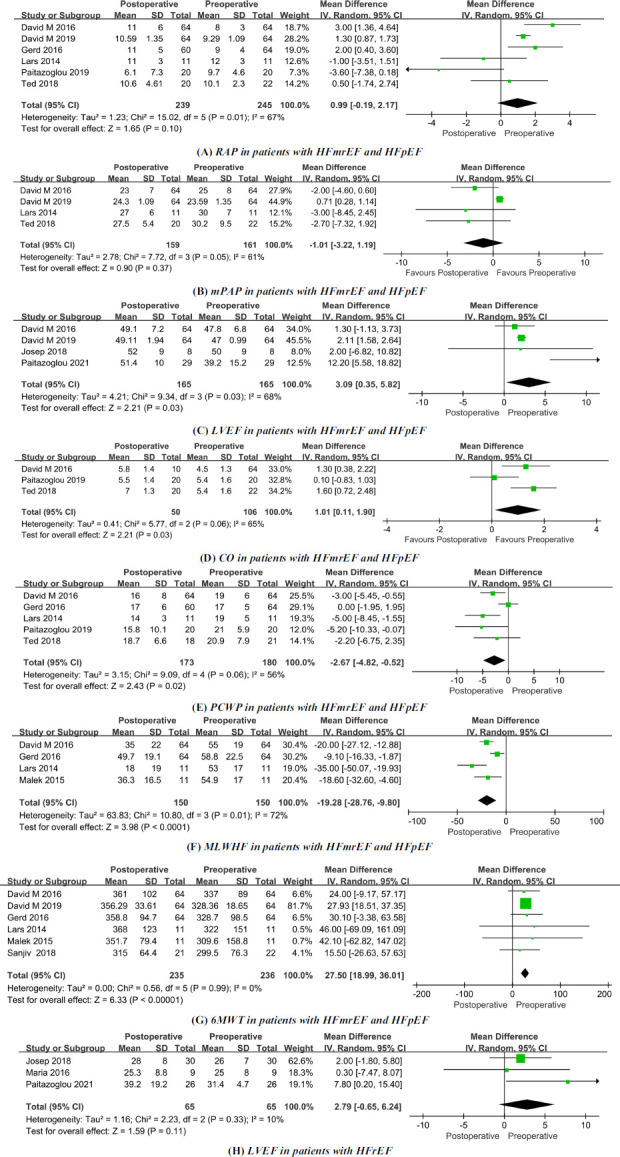
Results of subgroup analysis in patients with HFmrEF and HFpEF: (**A**) right atrial pressure (RAP), (**B**) mean pulmonary arterial pressure (mPAP), (**C**) left ventricular ejection fraction (LVEF), (**D**) cardiac output (CO), (**E**) pulmonary capillary wedge pressure (PCWP), (**F**) Minnesota Living with Heart Failure (MLWHF) score, (**G**) 6-minute walk test (6MWT). Results of subgroup analysis in patients with HFrEF: (**H**) left ventricular ejection fraction (LVEF).

The subgroup analysis outcomes for patients with HFpEF and HFmrEF revealed that there were no significant changes in RAP 0.99mmHg [95% CI (-0.19–2.17); I^2^=67%; *P*>0.0^5^] and mPAP [-1.01 mmHg [95% CI (-3.22,1.19); I^2^=61%; *P*>0.0^5^] post-operation (Figs. **4A** and **4B**). However, LVEF and CO exhibited significant increases after the procedure, with improvements of 3.09% [95% CI (0.35-5.82); I^2^=68%; *P*<0.0^5^] and 1.01 L/min [95% CI (0.11-1.90); I^2^=65%; *P*<0.0^5^] respectively (Figs. **4C** and **4D**). Furthermore, PCWP and MLWHF score demonstrated significant reductions after the operation, with changes of -2.67 mmHg [95% CI (-4.82 – -0.52); I^2^=56%; *P*<0.0^5^] and -19.28 [95% CI (-28.76 – -9.80); I^2^=72%; *P*<0.0^5^], respectively (Figs. **4E** and **4F**). Additionally, 6MWT distance achieved a significant increase of 27.5 meters [95% CI (18.99-36.01); I^2^=0%; *P*<0.0^5^] post-operation (Fig. **4G**). For patients with HFrEF, the implantation of IASD did not result in a significant increase of LVEF 2.79% [95% CI (-0.65-6.24); I^2^=10%; *P*>0.0^5^] (Fig. **4H**). Hemodynamic and echocardiographic measurements of subgroup results are shown in Table **S3**. Additionally, Table **S4** showed heterogeneity measures for all outcomes between groups.

### 3.4 Sensitivity Analysis

To assess the robustness of the findings, a sensitivity analysis was conducted by altering the statistical model to gauge potential changes in the results. The results of the sensitivity analysis are provided in Table **S5**.

### 3.5 Publication Bias

Begger's test and Egger's test were used to quantitatively detect publication bias. The *P*-values of Begger's and Egger's tests were greater than 0.05, suggesting no publication bias in the results (Table **S6**).

## 4 DISCUSSION

Heart failure is a common manifestation of heart disease, which can be caused by chronic structural or non-structural factors [46-48]. Despite great progress have been achieved for the treatment of the disease by novel medications and innovative devices, cardiovascular death, hospitalization rates and QoL remain unsatisfactory, especially in patients with HFpEF/HFmrEF [49-51]. Patients with heart failure typically experience clinical symptoms due to pulmonary vascular bed congestion caused by increased PCWP and LAP. Thus, devices designed to lower elevated LAP by creating a conduit from the left atrium to other chambers or structures emerge to be attractive. To date, 4 interatrial shunt devices are under clinical investigation, including Interatrial Shunt Device (IASD), V-Wave Shunt, Atrial Flow Regulator (AFR) and Transcatheter Atrial Shunt System [52-55]. Although the short-term effect of atrial septal shunt has been well-defined, the emergence of controversial results in this field raises concerns [24, 25, 28, 30]. The primary aim of this meta-analysis, which comprises a total of 13 studies involving 1026 participants, is to evaluate the efficacy of septal shunts in individuals with chronic heart failure.

Hemodynamic changes constitute the primary focus of study across all trials. In this meta-analysis, data from seven studies were analyzed, and it was found that the level of PCWP significantly decreased by 2.60 mmHg after atrial septal shunt implantation. Further subgroup analysis based on the type of HF showed that for patients with HFmrEF/HFpEF, atrial septal shunt implantation significantly decreased PCWP by 2.67 mmHg. Pulmonary artery congestion, secondary to increased PCWP, is the core mechanism of chronic heart failure [56]. Consistent with other analyses [57, 58], our results provided further evidence that atrial septal shunt effectively reduces the PCWP in all types of HF. The change of this parameter represents a significant improvement in exercise tolerance, augmenting quality of life, and clinical outcomes of HF patients. Cardiac output stands as a pivotal metric for evaluating cardiac function and holds the potential to serve as a predictive indicator for patient outcomes. Williams *et al*. found that peak cardiac output, as measured by cardiopulmonary exercise testing, was an independent predictor of mortality [59]. In the analysis of the effect of atrial septal shunt on cardiac output in patients with heart failure, 5 studies were included. The outcomes suggested that the cardiac function post-atrial septal shunt implantation did not exhibit a significant change when compared to their pre-operative state. However, sensitivity analysis implied that the results were unstable, possibly attributed to alterations of the statistical model and significant differences in cardiac output before and after the procedure. Consequently, a subgroup analysis was conducted according to the different types of HF, which revealed that atrial septal shunt significantly improved cardiac output in patients with HFmrEF/HFpEF. Another interesting revelation of this meta-analysis is that while LVEF significantly improved in HFmrEF/HFpEF patients, there is no significant increase in LVEF among patients with HFrEF. Ejection fraction, measuring the percentage of blood ejected during each heartbeat, has been the standard for assessing cardiac function but has significant limitations, particularly in HFpEF patients. Its variability due to factors like loading conditions, heart rate, and measurement techniques, combined with its inability to reflect diastolic function or myocardial strain, has prompted the search for alternative indices to better evaluate and manage heart failure [3, 60-62]. Thus, whether HFmrEF/HFpEF patients benefit from the improvement of LVEF needs to be further studied. PCWP is measured through invasive procedures and is less influenced by external factors compared to LVEF, providing more accurate results. It serves as a valuable hemodynamic parameter for assessing cardiac function and predicting prognosis in HF patients. In other HF hemodynamics, pulmonary diastolic pressure (PAD) can act as a surrogate for left-sided filling pressures, including LAPs [63]. Remote hemodynamic monitoring trials have shown positive results by reducing LAP, potentially offering greater accuracy than pulmonary arterial pressure monitoring [64]. Recent published REDUCE-LAP HF II and RELIEVE-HF results indicated that the efficacy of IASDs still remains controversial [24, 31]. One possible explanation is that part of the patients with HFmrEF and HFpEF enrolled in the studies had normal LA filling pressures at rest. This highlights the difficulty of diagnosing patients with symptomatic HF in this population when overt volume overload is not present at rest but symptoms with exertion are present. Thus, careful hemodynamic assessment with exercise may be needed. The V-LAP left atrium monitoring system has demonstrated safety and accuracy in measuring LA pressures, which may enhance outcomes for patients with HF [65, 66]. To date, Adona Medical has initiated a first-in-human trial of its adjustable interatrial shunt with bi-atrial pressure sensors for patients with HF. This technology offers the potential for more personalized treatments compared to existing shunt technologies.

Improvement of exercise intolerance and QoL are important in the evaluation of therapeutic goals in the treatment of HF patients. 6MWT is a widely used exercise test that is easy to conduct and cost-effective for evaluating the cardiac functional reserve of patients with chronic heart failure [67-71]. This test is often used to assess the effectiveness of treatment and has been shown to correlate with exercise capacity as measured by formal treadmill and cardiopulmonary exercise testing, as well as with morbidity and mortality in patients with HF [72-74]. In this study, we observed a significant average increase of 32.22 meters in 6MWT subsequent to atrial septal shunt implantation. Subgroup analysis also showed an increase of 27.50 meters among patients with HFmrEF/HFpEF, indicating a significant improvement in exercise endurance for patients with all types of HF. The Minnesota Living with Heart Failure Questionnaire (MLHFQ) is a comprehensive tool for evaluating the health-related quality of life in patients with heart failure [75-77]. Notably, a decline in MLHFQ scores has been established as a correlate to reduced mortality and decreased hospitalization rates [78]. In the analyses, we observed a consistent reduction in postoperative MLHFQ scores across all HF patients, including those with HFpEF/HFmrEF. Additionally, we also evaluated the Kansas City Cardiomyopathy Questionnaire (KCCQ-12) score for the patients with heart failure [79-81], which indicated that the QoL and exercise tolerance of patients were improved after atrial septal shunt implantation. Therefore, atrial septal shunt implantation holds the potential not only to enhance the quality of life but also to mitigate mortality and rehospitalization rates in patients with heart failure.

## 5 LIMITATIONS

Some limitations of our analysis should be acknowledged. First, current published trials are observational before-after studies and deficient in long-term follow-up data. It is important to notice that a more accurate evaluation of the potential pathophysiological advantages should be attained when considering an extended time frame. Second, detailed reporting of medical therapy management was lacking in most of the studies. Third, in an individual study [24], the sample size is relatively large, which may cause certain biases in the research results. Fourth, there may be confounding variables in the included studies, such as differences in follow-up time and population heterogeneity. Another limitation is that the results of 6MWT are always influenced by multiple factors, and cardiopulmonary function is just one of them. Thus, it could be valuable to apply a broader of assessment tools to evaluate exercise tolerance comprehensively.

## CONCLUSION

The implantation of atrial septal shunt devices increases right atrial pressure and LVEF in heart failure patients reduces PCWP, enhances exercise tolerance, and improves the overall quality of life. Most importantly, our subgroup analysis suggests that while atrial septal shunt implantation might not yield improvements in LVEF among individuals with HFrEF, it leads to enhancements in hemodynamic parameters, exercise endurance, and QoL among individuals affected by HFpEF/HFmrEF. We look forward to the publication of large-scale, high-quality, long-term randomized controlled trials to further clarify the effects of atrial septal shunts.

## Figures and Tables

**Table 1:** Basic characteristics of the studies.

**Study**	**Included Number**	**Age**	**Male Sex** **(%)**	**Device**	**Design**	**NYHA Class**	**LVEF**	**Follow-Up (Month)**
Sanjiv *et al.* (2022) [24]	626	73(67-77)	385(61.5%)	IASD	Randomized Controlled Trial	II–IV	≥40%	12
Paitazoglou *et al.* (2021) [27]	53	70(63-73)	31(59%)	AFR device	Prospective non-randomized study	III/IV	≥15%	12
Guimarães *et al.* (2020) [40]	6	68±9	NA	V-Wave	Non-randomized	III/IV	≥40%	12
Paitazoglou *et al.* (2019) [43]	36	67±9	21(58%)	AFR device	Prospective pilot study	III/IV	>15%	3
David *et al.* (2019) [28]	64	70±2	32(50%)	IASD	Single-arm, open-label study	II / III	>40%	6
Savjia *et al.* (2018) [29]	22	71(67-75)	14(64%)	IASD	Randomized clinical trial	III/IV	≥40%	12
Josep *et al.* (2018) [44]	38	69±9	35(92%)	V-Wave	Single-arm, open-label study	III/IV	—	12
Ted *et al.* (2018) [25]	22	70±8	14(64%)	IASD	Randomized Controlled Trial	III/IV	≥40%	1
David *et al.* (2016) [41]	64	69±9	NA	IASD	Prospective Single-arm study	II–IV	≥40%	12
Gerd *et al.* (2016) [30]	64	69±9	22(34%)	IASD	Prospective Single-arm study	II–IV	>40%	6
Maria *et al.* (2016) [39]	9	62±8	9(100%)	V-Wave	Cohort study	III/IV	<40%	3
Malek *et al.* (2015) [42]	11	70±12	6(55%)	IASD	Non-randomized	III/IV	≥45%	1
Lars *et al.* (2014) [45]	11	70±12	6(55%)	IASD	Pilot trial	III/IV	>45%	1

**Note: ** Data are mean±standard deviation, median (interquartile range).

**Table 2:** Hemodynamics, echocardiographic and QoL of the included studies.

**Study**	**RAP(mmHg)**	**LVEF(%)**	**CO (L/min)**	**mPAP** **(mmHg)**	**PCWP** **(mmHg)**	**6MWT(m)**	**MLWHF**	**KCCQ**
Sanjiv *et al.* (2022) [24]	--	--	--	--	--	--	--	45.8±23.5/ 57.6±24.8
Paitazoglou *et al.* (2021) [27]	--	--	--	--	--	200±50/250±37	--	--
Guimarães *et al.* (2020) [40]	--	34±12/38±9	--	--	--	274±65/338±104	--	51.1±9.7/ 83±15.4
Paitazoglou *et al.* (2019) [43]	10.5±5/11.7±7.6	41.9±11.6/46.5±13.6	5±1.6/4.8±1.6	--	20.5±5.5/ 16.6±8.5	217.9±113.2/ 245.9±26.1	--	--
David *et al.* (2019) [28]	9.3±1.1/10,6±1.4	47±1/49.1±1.9	--	23.6±1.4/24.3±1.1	--	328.4±18.7/ 356.3±33.6	--	--
Savjia *et al.* (2018) [29]	--	--	--	--	--	299.5±76.3/315±64.4	--	--
Josep *et al.* (2018) [44]	8±4/9±4	50±9/54±9	1.9±1/1.9±0.4	30±7/30±10	21±5/19±7	290±112/324±105	--	--
Ted *et al.* (2018) [25]	10.1±2/10.6±4.6	--	5.4±1.6/7±1.3	30.2±9.5/ 27.5±5.4	20.9±7.9/ 18.7±6.6	--	--	--
David *et al.* (2016) [41]	8±3/11±6	47.8±6.8/49.1±7.2	4.5±1.3/5.8±1.4	25±8/23±7	19±6/16±8	337±89/361±102	55±19/35±22	--
Gerd *et al.* (2016) [30]	9±4/11±5	--	--	--	17±5/17±6	328.7±98.5/ 358.8±94.7	58.8±22.5/ 49.7±19.1	--
Maria *et al.* (2016) [39]	9±4/8±5	24.5±8.3/25.3±8.8	4.4±1/5.5±0.9	29±7/26±11	23±5/17±8	244±112/319±134	--	44.3±9.8/ 79.1±13
Malek *et al.* (2015) [42]	--	--	--	--	--	309.6±158.8/ 351.7±79.4	54.9±17/ 36.3±16.5	--
Lars *et al.* (2014) [45]	12±3/11±3	--	--	30±7/27±6	19±5/14±3	322±151/368±123	53±17/18±19	--

**Note: ** Data are mean±standard deviation, preoperative/postoperative.

## Data Availability

The authors confirm that the data supporting the findings of this research are available within the article.

## LIST OF ABBREVIATIONS

6MWD: 6-minute Walking Distance
HF: Heart Failure
KCCQ: Kansas City Cardiomyopathy Questionnaire
LVEF: Left Ventricular Ejection Fraction
MLWHF: Minnesota Living with Heart Failure
mPAP: Mean Pulmonary Artery Pressure
RAP: Right Atrial Pressure

## STANDARDS OF REPORTING

PRISMA guidelines and methodology were followed.

## SUPPLEMENTARY MATERIAL

PRISMA checklist and supplementary material are available on the publisher’s website along with the published article.
